# A Comprehensive Study on The Accelerated Weathering Properties of Polypropylene—Wood Composites with Non-Metallic Materials of Waste-Printed Circuit Board Powders

**DOI:** 10.3390/ma12060876

**Published:** 2019-03-15

**Authors:** Shenghui Tian, Yuanfang Luo, Jizun Chen, Hui He, Yong Chen, Zhang Ling

**Affiliations:** 1 Chongqing Key Laboratory of Nano-Micro Composite Materials and Devices, School of Metallurgy and Materials Engineering, Chongqing University of Science and Technology, Chongqing 401331, China; tianshenghui@live.cn (S.T.); yongchen998@163.com (Y.C.); 2012024@cqust.edu.cn (Z.L.); 2 School of Materials Science and Engineering, Key Lab of Guangdong Province for High Property and Functional Macromolecular Materials, South China University of Technology, Guangzhou 510640, China; psyfluo@scut.edu.cn (Y.L.); chenjiz@esquel.com (J.C.)

**Keywords:** polypropylene, non-metallic materials of waste-printed circuit boards powders, wood polymer composites, oxidation induction time, UV resistance

## Abstract

In this study, non-metallic materials of waste-printed circuit board powders (WPCBP) were successfully used as reinforcing filler to produce polypropylene (PP)–wood composites, and their effect on the weathering properties of PP composites were fully evaluated via oxidation induction time (OIT), attenuated total reflectance Fourier-transform infrared spectroscopy (ATR-FTIR), differential scanning calorimetry, vicat softening point (VST), scanning electron microscopy, and mechanical properties analysis. The OIT analysis confirmed that the anti-thermal oxidative aging properties of PP–wood composites were decreased with the loading of WPCBP. Apart from that, the PP composite, reinforced with 30 wt.% of WPCBP, exhibited the highest value of active energy, which suggests that it is more sensitive to temperature and oxygen when compared with other PP composites. The mechanical properties analysis revealed that neat PP exhibited the poorest weathering properties after being subjected to UV exposure, and its retention rate of tensile strength and notched impact strength were only 70.6% and 59.6%, respectively, while WPCBP and wood flour (WF) could efficiently improve the retention rates of the mechanical properties of the PP composites when subjected to UV exposure. The visual appearance of the PP composites after being subjected to UV exposure showed more and smaller cracks with the loading of WPCBP and WF. The ATR-FTIR results revealed that the carbonyl index increased for all the weathered samples, and the more WPCBP was added into the PP composites led to a higher carbonyl index value, which might be due to the multivalent transition metals in WPCBP, which accelerate the photo-oxidation of the PP composites. The VST results show that both WPCBP and WF can effectively enhance the heat deformation resistance of the PP composites that have been subjected to UV exposure.

## 1. Introduction

With the rapid development of the electronic information industry, customers frequently update their electronic products, such as mobile phones, computers, televisions, etc. The average effective lifetime of these electronic products has decreased dramatically in recent years, producing a large amount of wasted electric and electronic equipment (WEEE) [1,2]. WEEE is one of the fastest growing types of solid waste, and nearly 65.4 million tons of WEEE were generated in 2017 according to the Solving the E-Waste Problem (StEP) initiative, which elevated WEEE to a global environmental issue and a social problem that our society must face [3]. Waste-printed circuit boards (WPCBs) are one of the major components in WEEE, and nearly 1.96 million tons of WPCB were predicted to be generated around the world [3,4]. 

WPCB could be divided into two parts, i.e., heavy metals and non-metallic materials (WPCBP). Heavy metals account for about 30wt. % of WPCB, and methods for the recovery of heavy metals have been widely investigated [5]. WPCBP consists of thermosetting resins, glass fibers, and other additives, and several methods have been developed in order to separate and recycle WPCBP, such as chemical, biological, hydrometallurgical, pyrometallurgical, mechanical, plasma, and organic solvent treatment methods [6]. However, there is still a lack of efficient and widely used recycling technologies, and such waste is usually treated by illegal land-filling in some Asian counties (such as Vietnam and China) or incineration due to its complex compositions [6,7]. Extensive studies have evaluated the possibilities of using WPCBP as a reinforcing filler in polymer composites, such as high-density polyethylene (HDPE) [8,9], polypropylene (PP) [10,11], polyvinyl chloride (PVC) [12], polyester [13,14], polyethylene terephthalate (PET) [15], etc. The achievement of high value-added reutilization of WPCBP in polymeric products is an important global issue, and it has drawn great attention in recent years due to economic and environmental concerns [16].

Wood–plastic composites (WPCs), also defined as wood–polymer composites, are made by blending plastic matrixes with agricultural or forestry waste wood fiber (WF), which gives the composite material a wood facade and many mechanical performance advantages [17,18]. WPCs have been more widely developed in recent years [19], being abundant, lightweight, formaldehyde-free, and moisture-proof with excellent dimensional stability [20,21], it have been widely used in the construction industry, garden architecture, decorative materials, and the automotive sector [22,23]. Weathering properties are critical for WPCs, because their performances are easily affected during outdoor applications [24], and the weathering properties of WPCs have been a subject of interest for many researchers, because WPCs are often chosen as decorative materials in exterior trim [25,26]. Recently, WPCBP has been selected as a reinforcing filler in polyolefin-based WPCs in order to lower material costs [8,27]. However, WPCBP contains residue metals, which cannot be completely recovered during the separating process, and the remaining toxic heavy metals might accelerate the degradation of polymer matrix chains and restrict the material’s potential industrial applications [8]. Moreover, there is little existing literature related to the accelerated weathering properties of PP composites reinforced by WPCBP. 

PP is one of the most widely used and fastest growing thermoplastics because of its several advantages with regard to cost and performance. However, as an engineering material, PP has the poor weathering properties and is sensitive to heat, light, and oxidation, which limits its industrial applications. Among various factors, photo-oxidation plays an important role in weathering, having remarkable effects on the mechanical and physical properties of materials that have been subjected to UV exposure. In the present work, the influence of WPCBP on the accelerated weathering properties of PP–wood composites were studied, and detailed mechanical, chemical, and thermal properties and structure characterizations were evaluated by oxidation induction time, Fourier- transform infrared spectroscopy, differential scanning calorimetry, vicat softening point, scanning electron microscopy, and mechanical properties analysis before and after UV exposure.

## 2. Experimental

### 2.1. Raw Materials

PP (PPH-T03) with a melt flow index (MFR) of 3.0 (+ 0.5) g/10 min at 200 °C was used in this investigation, and it was supplied by the Sinopec Maoming Company (Guangdong, China). WPCBP with a mesh of 80, which was provided by Qingyuan Jintian Co. (Guangdong, China), was selected for making PP composites. WPCBP was purified according to the reported method, it contained fiber particulate bundles, single-glass fibers, residue multivalent transition metals (such as Fe, Cu, and Ni), and thermosetting powders, and the particle size between 80 and 130 mm was the most prevalent of WPCBP which amounting to 36.4 wt% [8,27]. Maleated polypropylene (PP-g-MAH) with a grafting rate of 1.0% was obtained from Kingfa Scientific and Technological Co., Ltd (Guangdong, China). Recovered fir wood flour (WF) with a mesh of 80 was purchased from De Qinglin Wood Flour Co. (Zhejiang, China).

### 2.2. Experimental Procedure

#### 2.2.1. Preparation of Various PP Composites

The sample formulations of various PP composites are listed in Table 1. Prior to blending, WPCBP, WF, and PP-g-MAH were dried in a ventilated oven at 80 °C until a constant weight was reached. The melt mixing was done using a two-roll mill (XK-168, Lina Machinery (Dongguan) Industrial Co., Ltd., Guangdong, China) for 7 min at 160 (+3) °C. Then, the samples were compression molded (XLB-D, Zhejiang Hong Tu Machinery ManufacturingPlant, Jinhua, China) at 180 °C for 3 min, followed by the cold-press process at room temperature for 7 min before removing it from the machine. Finally, the samples were cut off for different tests according to the relatedAmerican Society for Testing Material (ASTM) standards.

#### 2.2.2. UV-Accelerated Weathering

The resistance properties of various PP composites to photo-degradation were tested after a cycle of exposure according to International Standardization Organization (ISO) 4892-1. All the PP samples were put into a UV-340A accelerated weatherometer (GOTECH Testing Machines Co., Ltd. Guangdong, China) the irradiance was fixed at 0.83, and the processing temperature was set to 50 °C for 15 days. After that, the samples were conditioned at room temperature for 24 h before the testing.

### 2.3. Testing and Characterization

#### 2.3.1. Oxidation Induction Time (OIT)

The OIT test was performed according to the ISO standard 11357-6:2002 using a differential scanning calorimeter (DSC) (TA Instruments, New Castle, USA) measurement (TA Q20). Firstly, the sample was heated from 30 °C to the test temperature (190 °C, 200 °C, 210 °C, 220 °C, and 230 °C, respectively) at a heating rate of 20 °C/min under nitrogen flow. Then, the sample was held in the test temperature for 5 min. Finally, the N_2_ flow was switched to oxygen, and the OIT curves were recorded. The OIT values were calculated by TA analysis software (TA Instruments, New Castle, USA).

#### 2.3.2. Mechanical Properties

Tensile tests were conducted with a Zwick/Roell Z010 universal mechanical testing machine (Zwick/Roell, Ulm, Germany) according to ASTM D638 standard, the test speed was fixed at 50 mm/min, and the tensile strength of PP composites before and after UV exposure were recorded. 

Impact testing with specimen dimensions of 80 mm × 12.7 mm × 4 mm was conducted according to ASTM D256 with a Zwick 5113 impact testing machine (Zwick/Roell, Ulm, Germany). In all cases, 5 specimens were tested, and the average values were reported.

In order to evaluate the UV resistance properties for various PP composites after UV exposure, the mechanical properties retention rates were calculated with the following formula (Equation (1)) in which S stands for variety strength, which includes tensile strength, flexural strength, and notched impact strength. It is generally recognized that composites cannot be used when their mechanical retention rates are less than 50% after weathering exposure.

(1) $$R\mathrm{etentionrate}=S_{afterUV\mathrm{exposure}}/S_{untreated\;samples}\times 100\%$$

#### 2.3.3. Optical Microscope (OM)

An optical microscope (OM) of OLYMPUS BX51(Olympus Corporation, Tokyo, Japan) was selected to observe the visual appearance of various PP composites after UV exposure, and the magnification was fixed at 200 times.

#### 2.3.4. Fourier-Transform Infrared (FTIR)

The PP composites before and after UV exposure were characterized using an attenuated total reflection (ATR) mode by a MAGBA-IR 760 spectrometer (Nicolet, Waltham, USA), and the wave number was set in the range of 4000–400 cm^−1^. The carbonyl index (CI) was calculated with the following formula (Equation (2)), in which A represents the peak area, A_2912_ corresponds to the peak area of C–H, and A_1715_ represents the peak area of C=O [28].

(2) CI=(A1715/A2912)×100%

#### 2.3.5. Differential Scanning Calorimetry (DSC)

A differential scanning calorimetry (TA instruments DSC Q20) (TA Instruments, New Castle, USA) test was also carried out to study the crystallization and melting behavior of the PP composites before and after UV exposure. The samples were heated at a rate of 10 °C/min from 30 °C to 190 °C under nitrogen and held for 3min. Then, the samples were dropped to room temperature at a rate of 10 °C/min. A total of 3 specimens of each formulation were tested, and the average values were reported.

T_p_ is defined as the crystallization peak temperature, while T_m_ is the melting peak temperature during the first heating cycle and T_m_–T_P_ is the degree of super cooling. ΔH_f_ is the melting enthalpy of the first heating cycle. The crystalline fraction (X_c_) of various PP composites can be determined from the followed equation (Equation (3)), in which f is the weight fraction of the filler, ΔH_f_ is the melting enthalpy of the sample, and ΔH_f_° is the theoretical melting enthalpy of 100% crystalline PP (209.0 J/g).

(3) $$Xc=[\Delta H_{f}/\Delta H_{f}{}^{\circ}(1-f)]\times 100\%$$

#### 2.3.6. Vicat Softening Point Analysis

The Vicat softening temperature (VST) of the PP composites before and after UV exposure was tested by Italian HDT-VICAT Thermal Deformation/VICAT Temperature tester (50 N loaded) (CEAST, Turin, Italy), and the heating rate was 120 °C/h. The temperature was defined as the VST when the needle was pressed 1 mm into the sample. 2 specimens of each formulation were tested (the difference is less than 1 °C) and the average values were calculated.

#### 2.3.7. Scanning Electron Microscopy (SEM)

A Quanta 200 scanning electron microscopy (SEM) machine [(FEI, Hillaboro, USA) was selected to study the impact fracture morphology of the PP composites (containing the PP matrix, WPCBP, and WF) before and after UV exposure, the SEM settings were listed as follows: high vacuum mode, sputtering Au (60 s), working distance of 10~13 mm, and an accelerating voltage of 10 kV.

## 3. Results and Discussion

### 3.1. OIT Values

**Figure 1:**
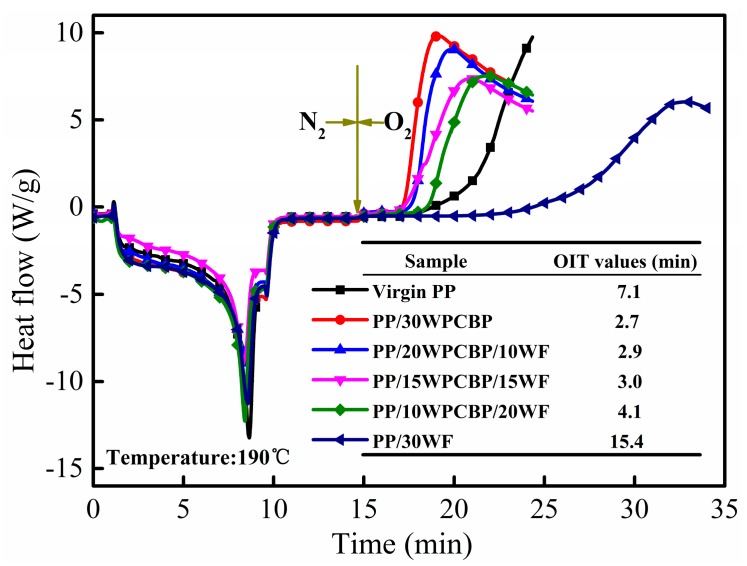
OIT curves and values of various PP composites.

OIT analysis has been widely used as an effective and easy index to assess the thermal oxidative aging properties of polymer composites [29]. Usually, a higher OIT value indicates that the sample has a better resistance to oxidative degradation [30,31]. The resistance to thermal oxidative aging of PP composites with different contents of WPCBP and WF was evaluated by OIT tests, as presented in Figure 1. The OIT value of virgin PP under 190 °C was 7.1 min, and it decreased dramatically with the increase of WPCBP in the PP composites (the total content of fillers was fixed at 30 wt.%). As can be seen clearly, the oxidation of PP/30WPCBP was detected immediately, and its OIT value (which was just 2.7 min) is much less than for virgin PP. This might due to the residue multivalent transition metals (such as Fe, Cu, and Ni) in WPCBP, which was proven to have an effect on accelerating the thermal oxidative degradation of PP molecular chains in our previous research [8]. Notably, it the addition of WF could increase the OIT values of the composites, indicating that WF had a positive significant contribution to the anti-thermal oxidative aging properties of the PP composites. This could be attributed to the component of lignin and hindered phenol in WF, which could act as capture agents for the living radicals and then delay the thermal oxidative degradation of the PP composites [18].

**Figure 2:**
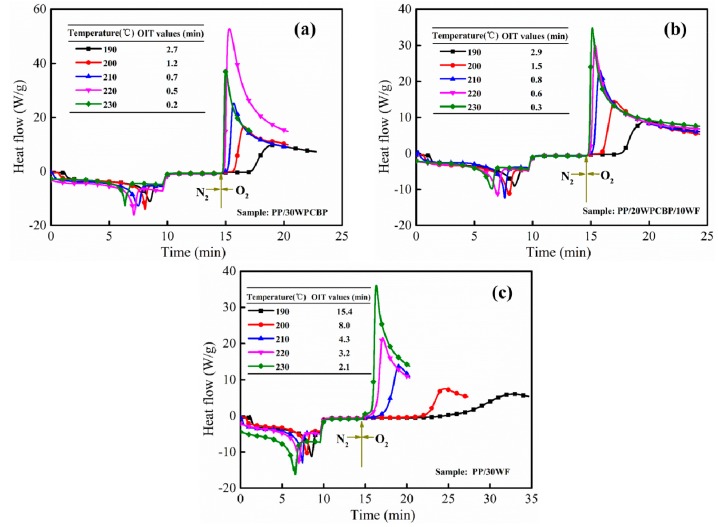
Oxidation induction time (OIT) curves for (**a**) PP/30WPCBP, (**b**) PP/20WPCBP/10WF, and (**c**) PP/30WF at different testing temperatures.

As was known, the OIT values of polymer composites were also influenced by the test temperature. Figure 2 and Table 2 showed the OIT curves and values of PP/30WPCBP (a), PP/20WPCBP/10WF (b) and PP/30WF (c) composites under different test temperatures. It was clearly seen that the OIT curves of three PP samples were left shifted and the OIT values decreased when increasing the test temperature [32]. The related OIT values of the above PP samples under different test temperatures were summarized in Table 2. It also should be noticed that there was a decrease in OIT values with the increase of WPCBP contents under the same test temperature, which could further demonstrate that WPCBP had a negative impact on the thermal oxidative ageing properties of PP composites.

**Figure 3:**
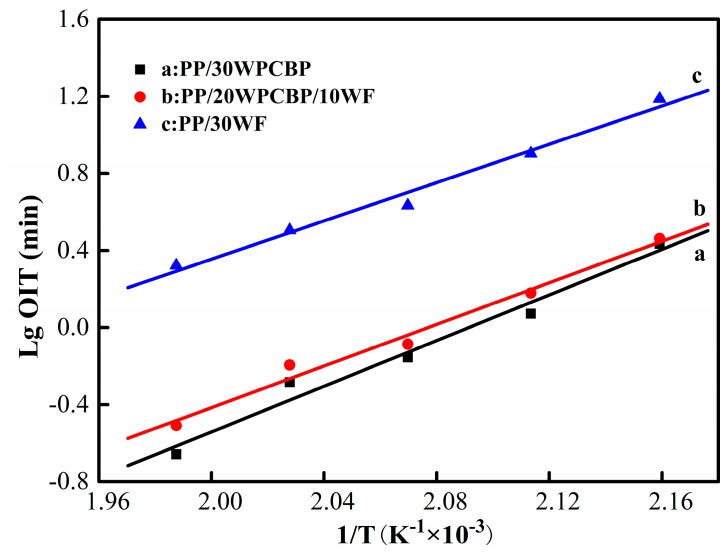
Fitting linear equations of different PP/WPCBP/WF composites.

The active energy (E) could be calculated by the OIT values from the linear relationship between the logarithm of OIT (Lg OIT) and the inverse of absolute test temperature (1/T) [31]. Usually, E reflects the sensitivity of the thermal oxidative aging of composites to temperature. A higher E represents poor thermal oxidative resistance for the composite, and its mechanical properties and surface luster are easily affected by the processing temperature [33]. The fitting linear equations of various PP/WPCBP/WF composites are shown in Figure 3, and the related activation energy data are summarized in Table 3. It was found out that the PP/30WPCBP composite had the highest value of E, which indicated that it was more sensitive to temperature than other PP composites, and it was a great negative factor to polymer processing [34,35]. With the increase of WF loading, the activation energy of the PP composite was decreased. This is consistent with the OIT values for the various samples.

### 3.2. Mechanical Properties

**Figure 4:**
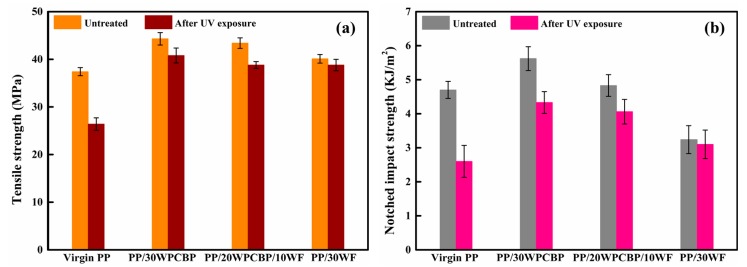
Tensile strength (**a**) and notched impact strength (**b**) of various PP composites before and after UV exposure.

Figure 4 shows the tensile strength and notched impact strength of virgin PP, PP/30WPCBP, PP/20WPCBP/10WF, and PP/30WF before and after UV exposure. As can be seen, the tensile strength of the PP composites was improved with the loading of WPCBP and WF, while the notched impact strength was increased with the constant loading of WPCBP. Apparently, the PP/30WPCBP composite showed the best mechanical properties among these samples. After 15 days of UV exposure, the tensile strength and notched impact strength for all the PP formulations decreased, and virgin PP showed the largest decreasing amount among them. Interestingly, the tensile strength values and notched impact strength values for all the accelerated PP composites containing WPCBP and WF were higher than virgin PP, and the PP/30WPCBP composite exhibited the highest tensile strength and notched impact strength among these samples.

The retention rate of tensile strength and notched impact strength of various PP samples after UV exposure are listed in Table 4. As can be seen, virgin PP exhibited the lowest retention rate of mechanical properties, and its retention rate of tensile strength and notched impact strength were only 70.6% and 59.6%, respectively, indicating that that virgin PP had the poorest UV resistance properties. It can also be observed from Table 4 that all the tensile strength retention rates and notched impact strength of the PP composites were increased with the loading of WPCBP and WF. Specifically, all the tensile strength retention rates of the PP/30WPCBP, PP/20WPCBP/10WF, and PP/30WF samples were higher than 90%, while the notched impact strength retention rate of the PP composites increased progressively with the increasing WF loading. These results are within expectations and are in agreement with the results obtained from the OIT values (which are shown in Figure 1 and Figure 3). All these data demonstrated that both WPCBP and WF could effectively improve the mechanical property retention rates of the PP composites after UV exposure, and WF is more pronounced than WPCBP.

### 3.3. Visual Appearance

**Figure 5:**
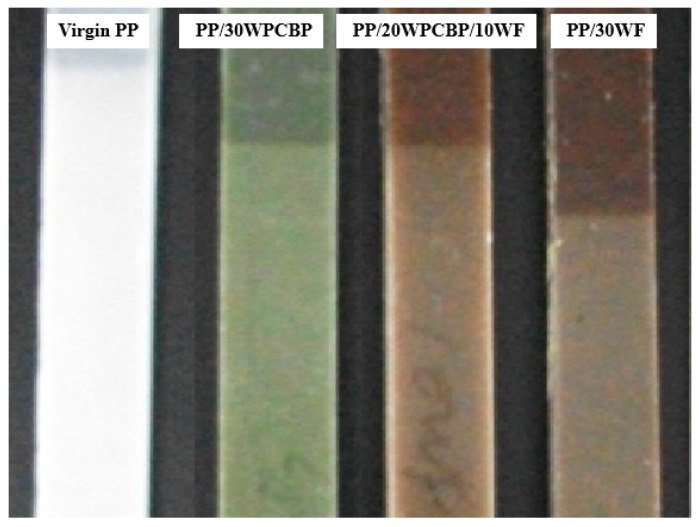
Visual photos of various PP composites after UV exposure.

**Figure 6:**
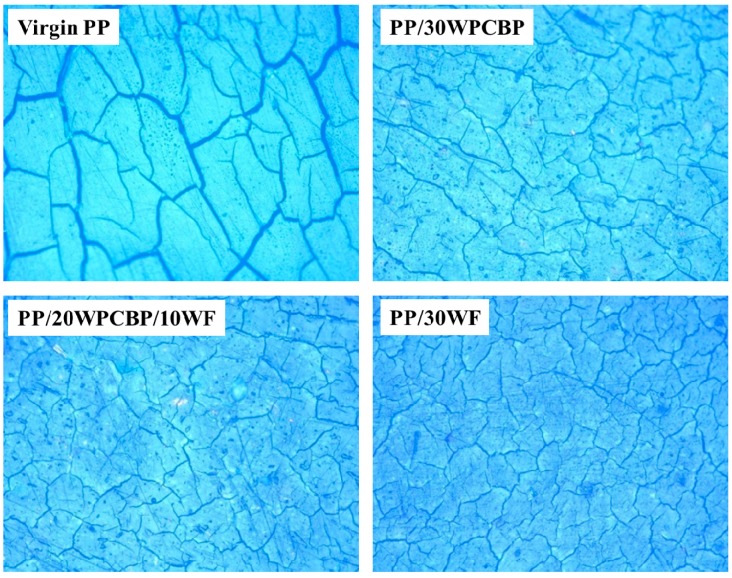
Digital photographs of various PP composites after UV exposure (× 200).

Polymers are subject to photo-ageing during UV exposure, and their visual appearance usually changes accordingly [36]. In this study, an optical microscope was used to study the visual appearance of the PP composites after UV exposure, and the obtained visual appearance and digital photographs (magnification: 200 times) are shown in Figure 5 and Figure 6. It is clear that the PP composites made from various formulations responded differently to UV exposure, but obvious cracks were found elsewhere in all the PP composites. The observed deep crisscrossed cracks (which are also known as crazing) demonstrated that the surface structure of virgin PP was destroyed dramatically during UV exposure [37]. It can also be seen from Figure 6 that with the loading of WPCBP and WF, the visual appearance of PP composites after UV exposure showed a tendency to develop increased short crack density and smaller cracks. Apart from that, the depth of the cracks was also decreased. These results further suggest that both WPCBP and WF can effectively improve the UV resistance properties of the PP composites.

### 3.4. Surface Chemistry

**Figure 7:**
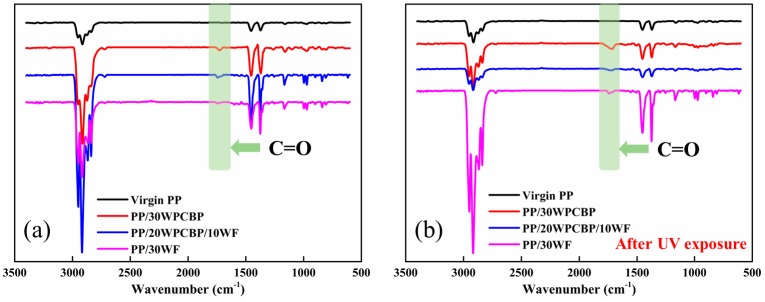
FTIR spectra of various PP composites (**a**) before and (**b**) after UV exposure.

It is well known that PP has poor weathering properties, as it contains a large amount of α-H, which is unstable and easily oxidized, and it is sensitive to heat, light, and oxidation, which hinders its outdoor applicability [38]. In this study, FTIR spectroscopy was adopted to evaluate the surface chemistry changes of various PP composites before and after UV exposure, and the carbonyl index (CI) calculated from the FTIR test could evaluate the oxidation of the composites. Usually, the more resistant a sample to UV degradation, the lower the CI value. The FTIR spectra of various PP composites (a) before and (b) after UV exposure are shown in Figure 7. 

It can be observed from Figure 6a that there were no obvious C=O peaks observed at 1715cm^−1^ in virgin PP before UV exposure, and the carbonyl groups (C=O peak) were observed with the loading of WPCBP and WF. This might be due to the fact that WPCBP consists of certain amounts of thermosetting plastics, and the lignin in WF is easily oxidized, generating carbonyl groups [39]. Carbonyl groups increased upon UV exposure, showing strong evidence of oxidation. It can be noted from Figure 6 that there is an increase in the intensities of the carbonyl groups at 1715 cm^−1^ after UV exposure, which indicates the occurrence of photo-oxidation in all the PP composites.

Table 5 summarized the relative carbonyl intensity of various PP samples before and after UV exposure, which were calculated from ATR-FTIR spectra. It can be observed that the PP/30WPCBP sample had the highest carbonyl intensities after UV exposure, indicating that the PP/30WPCBP sample was unstable when compared with other PP composites. Moreover, the observed increase in CI values after UV exposure also indicated that photo-oxidation occurred in all the PP composites during UV exposure and the more WPCBP added into the samples led to higher CI values. This result should be ascribed to the multivalent transition metals from WPCBP which could accelerate the photo-oxidation of the PP composites.

### 3.5. Morphology of the Fractured Surface

**Figure 8:**
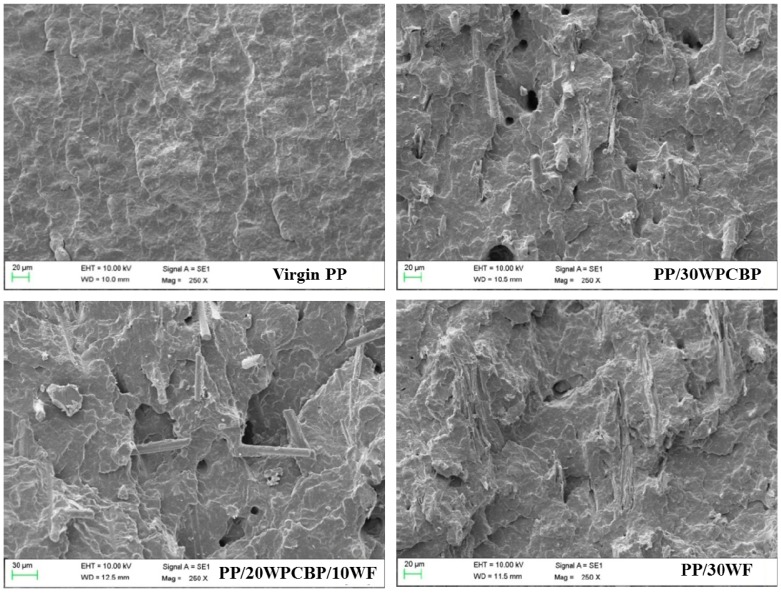
SEM photographs of various PP composites after UV exposure (× 250).

Figure 8 displays SEM photographs of the fractured surface of virgin PP and the reference PP composites after UV exposure (the magnification was fixed at 200 times). It can be seen that the impact fracture of virgin PP after 15 days of UV exposure was smoothed and dimmed, which showed a brittle rupture character. With the loading of WPCBP and WF, it also can be seen from Figure 8 that extensive fiber pull-out voids and debonding were found for the PP/30WPCBP composite after UV exposure, which was due to the degradation and notch-sensitivity of the accelerated composite and resulted in stress concentration more easily. Thus, the notched izod impact strength of the PP composite decreased. With the increase of WF, the pull-out holes decreased, which indicated that WF had better interfacial bonding with the PP matrix after UV exposure. It should be mentioned that the SEM results were within expectations and were also in good agreement with the results obtained from the OIT values and the notched izod impact strength retention rate, which is exhibited in Figure 1 and Table 4, respectively. 

### 3.6. DSC Analysis

**Figure 9:**
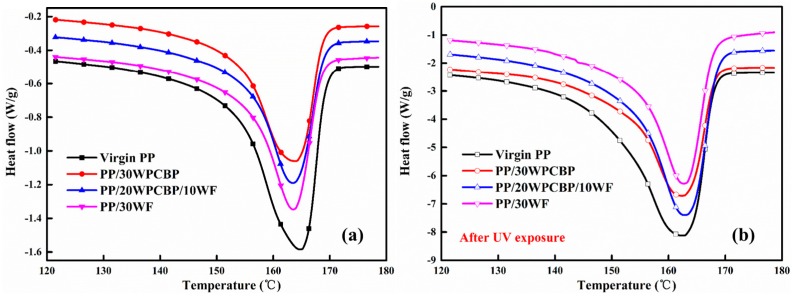
Differential scanning calorimetry (DSC) melting curves of PP composites (**a**) before and (**b**) after UV exposure.

**Figure 10:**
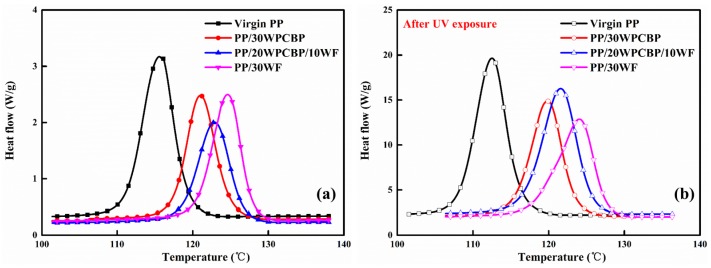
DSC crystallization curves of various PP composites(**a**) before and (**b**) after UV exposure.

As was known, virgin PP contains significant amounts of large size spherulites, which will lead to high molding shrinkage. In general, the incorporation of fillers can moderately reduce the molding shrinkage ratio of PP composites and improve their dimensional stability. In this study, DSC was selected to study the crystallization and melting behavior of various PP composites. Figure 9 and Figure 10 showed the melting curves and crystallization curves of various PP composites (a) before and (b) after UV exposure, respectively. The related data from DSC analysis were calculated and summarized in Table 6.

It can be observed that before UV exposure, the crystallization peak temperature T_P_ and the crystalline fraction X_c_ of various PP composites increased upon the incorporation of WPCBP and WF. The PP/30WF composite exhibited better crystallization behaviors than the other PP composites. Its T_P_ reached 124.6 °C, and X_c_ was 55.2%. This might be due to the heterogeneous nucleation effects of WPCBP and WF. As was known, the crystallization behavior of PP composites is affected by many factors, such as the composition, size of fillers, and distribution, etc. With the loading of WPCBP and WF, different types of heterogeneous nuclei activated, and homogeneous nucleation occurred, which could increase the X_c_ of the ensuring composites. As the average particle size and length of the diameter of WF was smaller than that of WPCBP in the same mesh, WF showed better nucleation effect on PP compared with WPCBP. It can also be seen from Table 6 that the degree of super cooling T_m_ − T_P_ decreased dramatically upon the incorporation of WF and WPCBP, which was beneficial to reduce the molding cycle times of PP composites [40]. In summary, incorporating WPCBP and WF into PP composites could efficiently improve the crystallization properties of the composites.

After UV exposure, all the PP samples showed decreases in TP, Tm, and Xc values. This might be due to the fact that the UV aging easily occurred in the non-crystalline region of the PP composites and accelerated the oxidative degradation of the PP molecule chains, which caused the mechanical properties (the tensile strength and notched impact strength) for all the PP formulations to decrease (Figure 4). However, the PP/30WF sample showed a very slight decrease compared to other PP composites, this might due to the fact that it was difficult to cause damage in the crystalline region in brief UV exposure and the WF fillers could absorb parts of the UV light, which could also alleviate the serious degradation of PP macromolecule chains.

### 3.7. Vicat Softening Temperature Analysis

The vicat softening temperature (VST) is defined as the temperature when the needle was pressed 1 mm into the sample under the application of a load (50 N), which is an important parameter to evaluate the heat resistance of the composite. Usually, the better the dimensional stability of the sample to the thermal deformation, the higher the VST value. 

**Figure 11:**
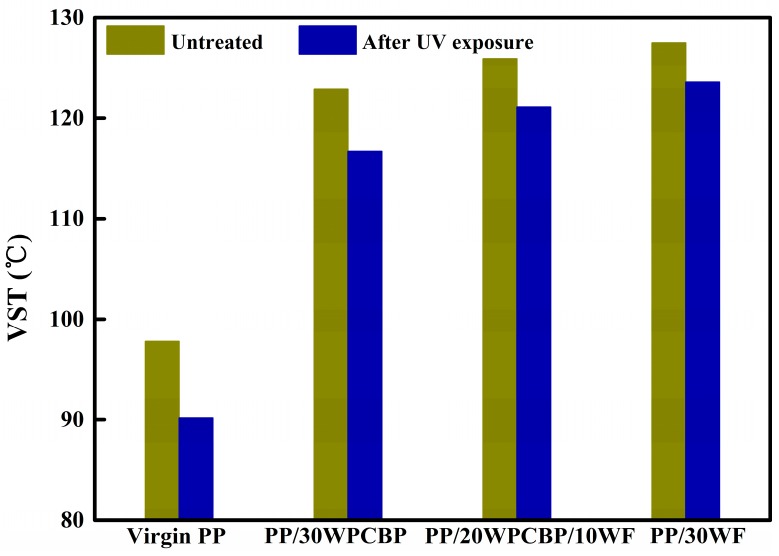
Vicat softening temperature (VST) values of various PP composites before and after UV exposure.

The VST values of various PP composites before and after UV exposure were investigated, and the results are summarized in Figure 11. Before UV exposure, the VST of virgin PP was 97.8 °C, and it could be observed that the VST of the PP composites increased dramatically with the loading of WPCBP and WF (all above 120 °C). This might due to the barrier effect caused by WPCBP and WF which had a load bearing high capability, then avoiding the stress concentration and resulting higher VST values of the composites. It can also be observed from Figure 11 that the VST values for all the PP composites decreased after UV exposure, especially those of virgin PP, which showed the sharpest drop in VST value, which was just 90.2 °C after UV exposure. It was clearly evident that WPCBP and WF can efficiently enhance the deformation resistance of PP composites

## 4. Conclusions

WPCBP was adopted to reinforce PP–wood composites, and its weathering properties were fully evaluated. OIT analysis confirmed that the anti-thermal oxidative aging properties of PP–wood composites decreased with the increase of WPCBP. Apart from that, the PP/30WPCBP composite had the highest value of active energy, which indicated that it was more sensitive to temperature than other PP composites. Virgin PP, PP/30WPCBP, PP/20WPCBP/10WF, and PP/30WF were weathered up to 15 days by being subjected to UV exposure. The mechanical properties analysis revealed that virgin PP exhibited the poorest weathering properties, with a tensile strength retention rate and a notched impact strength retention rate that were just 70.6% and 59.6%, respectively, and the incorporation of WPCBP and WF could remarkably improve the retention rates of the mechanical properties of the PP composites after UV exposure. OM results indicated that the visual appearance of the PP composites after UV exposure showed denser and smaller cracks with the loading of WPCBP and WF. ATR-FTIR results revealed that the carbonyl index increased for all the weathered PP samples; however, the more WPCBP that was added into PP composites led to a higher carbonyl index value, and this should be ascribed to the introduction of multivalent transition metals in WPCBP, which could accelerate the photo-oxidation of the PP composites. VST results suggested that WPCBP and WF can significantly enhance the deformation resistance of the PP composites. SEM analysis indicated that the fracture of virgin PP after UV exposure was smoothed and dimmed, which shown a brittle rupture character, and the fracture appearance changed with the loading of WPCBP and WF.

## Figures and Tables

**Table 1:** Sample formulations of various polypropylene (PP) composites. WPCBP: waste-printed circuit board powders; WF: wood flour; and PP-g-MAH: maleated polypropylene.

Sample	Composition (wt. %)			
	PP	WPCBP	WF	PP-g-MAH
Virgin PP	100	0	0	0
PP/30WPCBP	64	30	0	6
PP/20WPCBP/10WF	64	20	10	6
PP/15WPCBP/15WF	64	15	15	6
PP/10WPCBP/20WF	64	10	20	6
PP/30WF	64	0	30	6

**Table 2:** OIT values of various PP composites at different testing temperatures.

Sample	OIT (min)				
	190 °C	200 °C	210 °C	220 °C	230 °C
PP/30WPCBP	2.7	1.2	0.7	0.5	0.2
PP/20WPCBP/10WF	2.9	1.5	0.8	0.6	0.3
PP/30WF	15.4	8.0	4.3	3.2	2.1

**Table 3:** Activation energy of different PP/WPCBP/WF composites.

Sample	Lgτ=A/T+B	Activation Energy E = 2.303RA
PP/30WPCBP	Lgτ=5.921/T−12.38	113.3
PP/20WPCBP/10WF	Lgτ=5.921/T−11.21	103.3
PP/30WF	Lgτ=5.921/T−9.596	95.2

**Table 4:** Retention rate of mechanical properties of various PP composites after UV exposure.

Sample	Retention Rate (%)	
	Tensile Strength	Notched Impact Strength
Virgin PP	70.6	59.6
PP/30WPCBP	92.1	77.1
PP/20WPCBP/10WF	90.4	84.1
PP/30WF	96.8	95.7

**Table 5:** Relative carbonyl intensity (CI) of various PP samples before and after UV exposure.

Sample	A_2912_		A_1715_		CI	
	Before	After	Before	After	Before	After
Virgin PP	6.76	6.44	0	0.02	0	0.28
PP/30WPCBP	27.22	13.85	0.20	1.16	0.07	8.4
PP/20WPCBP/10WF	44.16	6.79	0.38	0.32	0.86	4.68
PP/30WF	21.02	28.16	0.13	0.34	0.62	1.2

**Table 6:** DSC data of various PP composites before and after UV exposure.

Sample	T_p_ (°C)		T_m_ (°C)		T_m_ − T_p_ (°C)		X_c_ (%)	
	Before	After	Before	After	Before	After	Before	After
Virgin PP	115.6	112.5	164.7	162.6	49.1	50.1	44.9	43.8
PP/30WPCBP	121.0	119.3	163.8	162.4	42.8	43.1	51.3	46.4
PP/20WPCBP/10WF	122.9	121.6	163.5	162.9	40.6	41.3	52.3	48.6
PP/30WF	124.6	123.1	163.6	162.8	39	39.7	55.2	53.2
